# Nutrition Knowledge, Information Sources, and Self-Perceived Ability to Provide Nutrition Counseling Among Medical Students: A Cross-Sectional Study

**DOI:** 10.3390/nu18193112

**Published:** 2026-09-22

**Authors:** Eva Luyufeng Yáñez-Izquierdo, Ana Presa-Carrera, Carlos Enrique Adán-Failde, Juan Manuel Vázquez-Lago

**Affiliations:** 1 Department of Preventive Medicine and Public Health, University of Santiago de Compostela, 15786 Santiago de Compostela, Spain; 2 Department of Preventive Medicine, Santiago de Compostela University Teaching Hospital, 15703 Santiago de Compostela, Spain; 3 Health Research Institute of Santiago de Compostela (IDIS), 15706 Santiago de Compostela, Spain

**Keywords:** nutrition knowledge, medical students, nutrition counseling, medical education, health promotion

## Abstract

Background/Objectives: Nutrition is an important component of health promotion and disease prevention, yet medical students may not feel sufficiently prepared to translate nutritional knowledge into patient counseling. This study aimed to assess nutrition knowledge among undergraduate medical students, identify factors associated with knowledge, and examine self-perceived ability to provide nutrition counseling and its associated factors. Methods: A cross-sectional study was conducted among medical students at the University of Santiago de Compostela, Spain, between February and April 2026. Nutrition knowledge and dietary and health-related behaviors were assessed using the refined 46-item version of a validated multidimensional questionnaire. Overall nutrition knowledge was calculated from 29 retained knowledge items. Bivariable analyses, multivariable linear regression, and multivariable logistic regression were performed. Results: A total of 413 students participated. Mean overall nutrition knowledge was 74.9 ± 14.4 points. Scores increased across academic stages, from 70.8 ± 13.6 in years 1–2 to 81.0 ± 13.3 in years 5–6 (*p* < 0.001). In the adjusted linear model, years 5–6 were associated with higher knowledge scores (β = 6.07; 95% CI 2.43–9.71; *p* = 0.001), as were reporting the medical curriculum (β = 3.42; 95% CI 0.29–6.54; *p* = 0.032) and media/social networks (β = 3.33; 95% CI 0.31–6.35; *p* = 0.031) as information sources. Overall, 37.3% of students considered themselves currently capable of providing nutrition counseling. In the adjusted logistic model, students in years 5–6 had greater odds of reporting current counseling ability (aOR = 3.20; 95% CI 1.63–6.29; *p* = 0.001), whereas women had lower odds of reporting current self-perceived counseling ability than men (aOR = 0.28; 95% CI 0.16–0.49; *p* < 0.001), despite no significant gender difference in objective nutrition knowledge. Healthier dietary and health-related behaviors were positively associated with counseling ability (aOR per 10-point increase = 1.63; 95% CI 1.21–2.20; *p* = 0.001), while overall nutrition knowledge was not independently associated with it (*p* = 0.437). Conclusions: Students in later academic years had higher nutrition knowledge scores, but greater knowledge did not independently translate into greater self-perceived counseling ability. These findings support longitudinal, practical, and competency-based nutrition education that addresses both knowledge acquisition and counseling preparedness.

## 1. Introduction

Diet is a major modifiable determinant of health and an important component of the prevention and management of noncommunicable diseases [1]. Physicians are therefore expected to contribute to health promotion and to provide evidence-based nutrition advice as part of routine clinical care. However, effective nutrition care requires adequate scientific knowledge, the ability to translate dietary recommendations into practical advice, communication skills, and confidence in counseling patients. Despite the importance of these competencies, nutrition education remains heterogeneous and frequently insufficient across medical curricula [2,3]. Studies involving medical students have consistently identified gaps in nutrition knowledge, practical skills, and confidence in delivering nutrition care, including among students approaching graduation [2,4]. Accordingly, recent international and multidisciplinary consensus initiatives have emphasized the need to define and integrate explicit nutrition competencies throughout undergraduate and postgraduate medical education [5,6].

Nutrition competence extends beyond knowledge of nutrients and general dietary recommendations. Future physicians need to understand the nutritional composition of commonly consumed foods, interpret food-related information, critically evaluate dietary claims, and communicate evidence-based recommendations in ways that are meaningful to patients [5,6,7]. These skills are closely related to the broader concepts of nutrition and food literacy, which encompass the ability to access, understand, evaluate, and apply nutrition information [7]. This is particularly relevant in an increasingly complex information environment in which students obtain nutrition-related content from formal curricula and scientific literature, but also from the Internet, media, social networks, family, and peers [8]. Frequently consulted information sources are not necessarily those considered most trustworthy [8], and nutrition-related misinformation is widely disseminated through social media platforms [9]. At the same time, educational evidence indicates that nutrition knowledge, personal health behaviors, self-efficacy, and preparedness to provide nutrition care are related but distinct outcomes. Nutrition education interventions can improve knowledge, behaviors, and counseling self-efficacy, but these dimensions do not necessarily change to the same extent [10,11]. Consequently, assessing factual and applied knowledge together with information sources and perceived counseling ability may provide a more comprehensive picture of nutrition-related educational needs among medical students.

Accurate assessment of these dimensions also requires instruments that are appropriate for the population being studied. A recently developed validated multidimensional questionnaire specifically designed for medical students integrates general nutrition knowledge, food composition knowledge, and dietary habits, providing an instrument for identifying specific educational gaps rather than relying exclusively on generic nutrition knowledge measures [12]. Using the refined version of this instrument, the present study aimed primarily to assess nutrition knowledge among undergraduate medical students and examine differences across stages of medical training. Secondary aims were to identify sociodemographic, academic, behavioral, and informational factors associated with nutrition knowledge; assess students’ self-perceived ability to provide nutrition counseling and factors associated with this perception; and identify specific areas of lower nutrition and food composition knowledge that could inform future educational strategies.

## 2. Materials and Methods

### 2.1. Study Design and Setting

A cross-sectional observational study was conducted among undergraduate medical students at the University of Santiago de Compostela (USC), Spain. Data collection took place between February and April 2026. The study was designed to assess nutrition knowledge, food composition knowledge, dietary and health-related behaviors, sources of nutrition information, and self-perceived ability to provide nutrition counseling across different stages of undergraduate medical training.

The study is reported in accordance with the Strengthening the Reporting of Observational Studies in Epidemiology (STROBE) recommendations for cross-sectional studies [13].

### 2.2. Study Population and Participant Recruitment

The target population comprised students enrolled in the six-year undergraduate medical degree program at the USC during the 2025–2026 academic year. Students were eligible to participate if they were aged 18 years or older, were enrolled in any of the six academic years of the medical degree, and voluntarily agreed to participate. Students younger than 18 years and those who did not provide consent were excluded.

Participants were recruited using non-probability convenience sampling. The questionnaire was administered electronically through Microsoft Forms (Microsoft Corporation, Redmond, WA, USA) and distributed to students from all six academic years through an electronic link and QR code disseminated through student communication channels and in classrooms.

Before accessing the questionnaire, participants received information about the objectives of the study, the voluntary nature of participation, the anonymity of data collection, and the academic and research purposes for which the information would be used. No personally identifying information was collected. Completion and submission of the questionnaire constituted informed consent.

### 2.3. Data Collection Instrument

Data were collected using a previously validated multidimensional questionnaire designed to assess nutrition knowledge, food composition knowledge, and dietary habits in medical students [12]. The questionnaire included an initial section collecting sociodemographic, academic, and contextual information, including age, gender, height, weight, academic year, place and duration of residence, household income, presence of relevant chronic conditions, adherence to a specific diet or dietary pattern, supplement use during the previous six months, sources of nutrition information, and self-perceived ability to provide nutrition counseling.

Participants could report up to three main sources of nutrition information from the following predefined categories: the medical degree curriculum as a source of nutrition information; curricula from other university degrees or vocational training programs; non-formal education; scientific literature; media and social networks; family, friends, or other people in the immediate social environment; individual advice from a professional; no previous nutrition training; or other sources. Each predefined source was subsequently coded as a separate binary variable for statistical analysis.

Self-perceived ability to provide nutrition counseling was assessed using three response categories: (1) currently capable; (2) not yet capable, but expected to be capable after completing the degree; and (3) not expected to be capable even after graduation.

In addition to the sociodemographic and contextual section, the questionnaire comprised three domains: general nutrition knowledge, food composition knowledge, and dietary habits: 11 general nutrition knowledge items, 18 food composition knowledge items, and 17 dietary habit items [12].

The general nutrition knowledge domain comprised 11 statements addressing general dietary recommendations and nutrition-related concepts. Responses were recorded using a five-point Likert-type scale ranging from “totally disagree” to “totally agree” and were classified as correct or incorrect according to the published scoring criteria [12]. The food composition knowledge domain included 18 items assessing participants’ ability to identify the nutritional characteristics of commonly consumed foods, including their salt, added-sugar, protein, and fiber content. Responses were scored as correct or incorrect according to the validated scoring key [12]. The dietary habits domain comprised 17 retained items assessing dietary practices and related health behaviors. Items were coded so that higher values consistently represented healthier behaviors, with reverse coding applied when necessary. For the present study, these items were combined into a dietary and health-related behaviors score, re-scaled from 0 to 100, with higher values representing a more favorable behavioral profile.

### 2.4. Study Variables and Outcomes

The primary outcome was the overall nutrition knowledge score, calculated using the 29 knowledge items retained in the refined version of the questionnaire, comprising 11 general nutrition knowledge items and 18 food composition knowledge items.

The overall score was calculated as follows: overall nutrition knowledge score = (number of correct responses across the 29 retained knowledge items/29) × 100. Higher scores indicated greater overall nutrition knowledge.

Secondary outcomes included the general nutrition knowledge score, food composition knowledge score, dietary and health-related behaviors score, and self-perceived ability to provide nutrition counseling. The dietary and health-related behaviors score was calculated from the 17 behavioral items retained in the refined questionnaire. All 17 retained behavioral items contributed equally to the composite score. Each item was scored from 0 to 4 and oriented so that higher values represented healthier behavior; negatively oriented items were reverse-coded. The summed score was rescaled to a 0–100 range. The composite was intended to represent an overall dietary and health-related behavioral profile rather than any individual behavior. Item-level percentages of correct responses were calculated exclusively for the 29 retained knowledge items to identify specific areas of lower performance.

For the main analyses, academic progression was categorized into three stages: years 1–2, years 3–4, and years 5–6, with years 1–2 used as the reference category in multivariable analyses.

Household income was categorized as low/medium–low (<EUR 2100 per month), medium–high (EUR 2100–3600), high (>EUR 3600), and unknown/prefer not to answer.

For the analysis of self-perceived counseling ability, the original three-category variable was dichotomized as currently capable versus not currently capable. The latter category combined students who did not yet consider themselves capable but expected to become capable after graduation and those who did not expect to become capable after completing the degree. This categorization was selected because the outcome of interest was the participant’s perception of their current self-perceived ability to provide nutrition counseling.

### 2.5. Statistical Analysis

Descriptive analyses were performed for the main study variables. Categorical variables were summarized as absolute frequencies and percentages, whereas continuous variables were expressed as means and standard deviations (SDs) and, where appropriate, medians and interquartile ranges (IQRs).

All questionnaire-derived scores were calculated using the 46 items retained in the refined version of the validated instrument: 11 general nutrition knowledge items, 18 food composition knowledge items, and 17 dietary and health-related behavior items. Overall nutrition knowledge was therefore calculated from the combined 29 retained knowledge items [12].

Bivariable analyses were conducted to examine associations between overall nutrition knowledge and sociodemographic, academic, behavioral, and informational variables. Student’s *t*-test was used for comparisons between two groups, whereas one-way analysis of variance (ANOVA) was used for variables with more than two categories. The association between age and overall nutrition knowledge was assessed using Spearman’s rank correlation coefficient. No adjustment for multiple comparisons was applied to the exploratory bivariate and item-level analyses. Their *p*-values should therefore be interpreted as exploratory rather than confirmatory. The principal inferential analyses were based on the prespecified multivariable regression models.

A multivariable linear regression model was fitted with the continuous overall nutrition knowledge score as the dependent variable. Covariates included gender, age, academic stage, household income, adherence to a specific diet or dietary pattern, supplement use during the previous six months, and the predefined sources of nutrition information. The non-specific “other source” category was excluded because of its very low frequency. Covariates were specified a priori according to their conceptual relevance to the study objectives and were retained in the model irrespective of their statistical significance in bivariable analyses. Heteroscedasticity-robust standard errors were used. Results were expressed as adjusted β coefficients with 95% confidence intervals (95% CIs) and *p*-values. Model fit was summarized using R^2^ and adjusted R^2^.

A second multivariable model was fitted to identify factors associated with current self-perceived ability to provide nutrition counseling. Binary logistic regression was used, with currently capable coded as 1 and not currently capable coded as 0. This outcome was dichotomized as currently capable versus not currently capable because the primary interest was students’ perception of their present counseling ability. Covariates included gender, age, academic stage, household income, adherence to a specific diet or dietary pattern, supplement use, the 17-item dietary and health-related behaviors score, the 29-item overall nutrition knowledge score, and the predefined sources of nutrition information. Overall nutrition knowledge and the dietary and health-related behaviors score were entered per 10-point increase to facilitate interpretation. Results were expressed as adjusted odds ratios (aORs), 95% CIs, and *p*-values. Model fit was evaluated using McFadden’s pseudo-R^2^, and discriminatory ability was assessed using the area under the receiver operating characteristic curve (AUC).

For the linear regression model, residual-versus-fitted and Q-Q plots, studentized residuals, leverage, and Cook’s distance were examined to assess model behavior and potentially influential observations. For the logistic regression model, convergence, influential observations, and the adequacy of the linear-in-the-logit specification for continuous predictors were examined. No major model-specification problems were identified.

The three participants identifying as non-binary or another gender category were retained in descriptive analyses but excluded from multivariable models because the small subgroup size did not permit stable gender-specific estimates.

No missing values were imputed. Complete-case analysis was used for the primary multivariable models. For the counseling model, the primary analysis required complete responses to the 17 retained behavioral items used to calculate the dietary and health-related behaviors score. A sensitivity analysis was additionally performed using this score calculated from the available retained behavioral items.

Multicollinearity was assessed using variance inflation factors (VIFs). Statistical significance was defined as a two-sided *p* < 0.05.

All analyses were conducted using Python version 3.13.5. Data management and numerical operations were performed using pandas version 2.2.3 and NumPy version 2.3.5; bivariable statistical analyses were conducted using SciPy version 1.17.0; linear and logistic regression models and model diagnostics were performed using statsmodels version 0.14.6; and the area under the receiver operating characteristic curve was calculated using scikit-learn version 1.8.0. openpyxl version 3.1.5 was used to import the Microsoft Excel dataset.

### 2.6. Ethical Considerations

The study protocol was reviewed and approved by the Santiago-Lugo Research Ethics Committee on 23 July 2025 (registration code 2025/320). Participation was voluntary and data were collected anonymously. Before completing the questionnaire, participants received information about the study objectives, the anonymous nature of data collection, the intended academic and research use of the information obtained, and their freedom not to participate. Completion and submission of the questionnaire constituted informed consent.

## 3. Results

### 3.1. Participant Characteristics

A total of 413 medical students participated in the study, corresponding to an estimated participation rate of 25.7%. Mean age was 21.7 ± 3.23 years. Most participants were women (76.8%), and 41.2% were enrolled in years 1–2, 31.7% in years 3–4, and 27.1% in years 5–6. Based on calculated body mass index, 78.0% of participants were within the normal-weight range.

Thirty-three participants (8.0%) reported following a specific diet or dietary pattern, and 161 (39.0%) had used dietary supplements during the previous six months. Overall, 154 students (37.3%) considered themselves currently capable of providing nutrition counseling, whereas 200 (48.4%) did not yet feel capable but expected to be so after graduation, and 59 (14.3%) did not expect to be capable even after completing the degree.

Media and social networks were the most frequently reported sources of nutrition information (64.2%), followed by the medical degree curriculum (49.9%), family, friends, or the immediate social environment (42.6%), and scientific literature (36.1%) (Table 1).

### 3.2. Nutrition Knowledge and Dietary and Health-Related Behaviors

All 413 participants had complete responses for the 29 knowledge items retained in the refined questionnaire. The mean general nutrition knowledge score, calculated from the 11 retained items, was 72.7 ± 17.4 points, with a median of 72.7 (IQR 63.6–81.8). The mean food composition knowledge score, based on the 18 retained items, was 76.2 ± 16.1 points, with a median of 77.8 (IQR 66.7–88.9).

The resulting overall nutrition knowledge score, calculated from all 29 retained knowledge items, was 74.9 ± 14.4 points, with a median of 75.9 (IQR 65.5–86.2).

The 17-item dietary and health-related behaviors score could be calculated from available responses for 411 participants. Its mean value was 73.7 ± 9.8 points, with a median of 73.5 (IQR 66.2–80.9).

### 3.3. Bivariate Associations with Overall Nutrition Knowledge

Overall nutrition knowledge differed significantly across academic stages. Mean scores increased from 70.8 ± 13.6 among students in years 1–2 to 75.0 ± 14.7 in years 3–4 and 81.0 ± 13.3 in years 5–6 (*p* < 0.001).

Students following a specific diet or dietary pattern had higher mean knowledge scores than those who did not (82.9 ± 12.4 vs. 74.2 ± 14.4; *p* < 0.001). Higher scores were also observed among students reporting supplement use during the previous six months (77.6 ± 13.5 vs. 73.1 ± 14.8; *p* = 0.002).

Knowledge differed according to self-perceived counseling ability (*p* < 0.001), with the highest mean score among students who considered themselves currently capable of providing nutrition counseling (79.1 ± 14.8).

Students reporting the medical curriculum, scientific literature, or media and social networks as nutrition information sources had significantly higher knowledge scores. Conversely, students reporting the immediate social environment as a source and those reporting no previous nutrition training had lower scores. No statistically significant differences were identified according to gender, household income, professional advice, non-formal education, or curricula from other degrees or training programs. Age showed a weak positive correlation with overall nutrition knowledge (Spearman’s ρ = 0.220; *p* < 0.001). See Table 2.

### 3.4. Factors Independently Associated with Overall Nutrition Knowledge

The multivariable linear regression model included 410 participants (Table 3). After adjustment for sociodemographic characteristics, academic stage, dietary characteristics, supplement use, and sources of nutrition information, students in years 5–6 had an overall nutrition knowledge score 6.07 points higher than those in years 1–2 (adjusted β = 6.07; 95% CI 2.43–9.71; *p* = 0.001). The difference between years 3–4 and years 1–2 was not statistically significant after adjustment (β = 2.34; 95% CI −1.01 to 5.69; *p* = 0.171).

Reporting the medical degree curriculum as a source of nutrition information was independently associated with a 3.42-point higher knowledge score (95% CI 0.29–6.54; *p* = 0.032). Reporting media and social networks was also associated with a higher adjusted score (β = 3.33; 95% CI 0.31–6.35; *p* = 0.031).

Although adherence to a specific dietary pattern and supplement use were associated with higher knowledge in the bivariable analyses, neither remained statistically significant after multivariable adjustment (dietary pattern: β = 4.50; *p* = 0.076; supplement use: β = 2.86; *p* = 0.058). No independent associations were observed for gender, age, household income, scientific literature, immediate social environment, professional advice, non-formal education, absence of previous nutrition training, or curricula from other degrees or training programs.

The model explained 18.6% of the variance in overall nutrition knowledge (R^2^ = 0.186; adjusted R^2^ = 0.151). No relevant multicollinearity was detected (maximum VIF = 1.87).

### 3.5. Self-Perceived Ability to Provide Nutrition Counseling

The proportion of students who considered themselves currently capable of providing nutrition counseling increased across academic stages: 41 of 170 students in years 1–2 (24.1%), 45 of 131 in years 3–4 (34.4%), and 68 of 112 in years 5–6 (60.7%).

The primary multivariable logistic regression included 397 participants with complete data for the 17 retained items contributing to the dietary and health-related behaviors score. Of these, 148 reported currently feeling capable of providing nutrition counseling (Table 4).

After adjustment, students in years 5–6 had more than three times the odds of reporting current counseling ability compared with those in years 1–2 (aOR = 3.20; 95% CI 1.63–6.29; *p* = 0.001). No statistically significant difference was observed between years 3–4 and years 1–2.

Women had lower odds of reporting current counseling ability than men (aOR = 0.28; 95% CI 0.16–0.49; *p* < 0.001). A higher dietary and health-related behaviors score was independently associated with greater perceived counseling ability: each 10-point increase was associated with 63% higher odds of considering oneself currently capable (aOR = 1.63; 95% CI 1.21–2.20; *p* = 0.001).

Overall nutrition knowledge was not independently associated with current self-perceived counseling ability after adjustment (aOR per 10-point increase = 1.08; 95% CI 0.89–1.30; *p* = 0.437). None of the individual sources of nutrition information showed a statistically significant independent association with this outcome.

The model had a McFadden pseudo-R^2^ of 0.179 and an AUC of 0.776. No relevant multicollinearity was identified (maximum VIF = 1.90).

A sensitivity analysis using the dietary and health-related behaviors score calculated from the available retained behavioral items included 408 participants. The main findings remained materially unchanged: women had lower odds of reporting current counseling ability (aOR = 0.30; 95% CI 0.17–0.52; *p* < 0.001), students in years 5–6 had higher odds than those in years 1–2 (aOR = 3.33; 95% CI 1.70–6.53; *p* < 0.001), and the dietary and health-related behaviors score remained positively associated with the outcome (aOR per 10 points = 1.66; 95% CI 1.23–2.24; *p* < 0.001). Overall nutrition knowledge remained non-significant (aOR = 1.08; 95% CI 0.90–1.30; *p* = 0.417).

### 3.6. Specific Nutrition Knowledge Gaps

The lowest proportion of correct responses was observed for the classification of red meat as having a high salt content, for which 22.3% correctly disagreed with the statement. Other areas of lower performance concerned the calcium content of whole versus skimmed milk (40.2% correct), gluten content of cereals (48.4%), salt content of frozen vegetables (55.9%), and the usefulness of approximate portion-size techniques such as the hand method (56.2%) (Table 5).

## 4. Discussion

The present study examined nutrition knowledge, information sources, dietary and health-related behaviors, and self-perceived ability to provide nutrition counseling among medical students. Three main findings emerged. First, students in years 5–6 had higher nutrition knowledge scores than those in years 1–2 after multivariable adjustment. Second, reporting the medical curriculum and media/social networks as sources of nutrition information was independently associated with higher knowledge. Third, although self-perceived counseling ability was more frequent among students in later academic years, overall nutrition knowledge was not independently associated with this perception. These findings support considering nutrition knowledge and perceived counseling preparedness as related but distinct educational outcomes.

Students in years 5–6 scored approximately six points higher than those in years 1–2, consistent with previous studies showing that greater exposure to nutrition-related content during medical education may be associated with better knowledge and preparedness [2,3,4]. Reporting the medical curriculum as a source of nutrition information was also independently associated with higher scores. However, this finding should not be interpreted as evidence of curricular effectiveness, since actual exposure to nutrition education, including teaching hours, content, methods, and clinical integration, was not measured. Moreover, the cross-sectional design compares different student cohorts rather than longitudinal changes within the same individuals. The educational significance of this difference should be interpreted cautiously. The study was cross-sectional, academic stages represented different student cohorts, and the model explained a modest proportion of the overall variance in nutrition knowledge. Therefore, the observed difference should not be interpreted as a direct measure of longitudinal curricular effectiveness. Nevertheless, the results are consistent with current recommendations that nutrition competencies should be integrated longitudinally throughout medical training [5].

Item-level findings identified difficulties related to food composition and practical nutrition concepts, including salt content, calcium, gluten, and portion-size estimation. Some of these items may depend on product characteristics, processing, or precise wording and should therefore be interpreted as areas requiring further assessment rather than definitive misconceptions. From an educational perspective, however, they may help identify topics for targeted training. Practical activities involving food composition, food-label interpretation, comparison of food products, common nutrition misconceptions, and portion estimation could complement theoretical instruction and facilitate translation of knowledge into patient-centered advice [5].

Media and social networks were the most frequently reported source of nutrition information and were independently associated with higher knowledge. This association requires careful interpretation. Because these sources were assessed as a single combined category, the study cannot distinguish between platforms, types of content, frequency of use, credibility, or scientific quality. Consequently, the finding does not indicate that social media improves nutrition knowledge. Previous research has shown that frequently consulted information sources are not necessarily those perceived as most trustworthy [8], while nutrition-related misinformation remains common in digital environments [9]. The high use of these sources nevertheless highlights the relevance of incorporating critical appraisal of nutrition information into medical education.

Only 37.3% of students considered themselves currently capable of providing nutrition counseling, although this proportion was substantially higher in years 5–6. Importantly, nutrition knowledge was not independently associated with self-perceived counseling ability after adjustment. Similar discrepancies between knowledge and confidence have been reported previously [14], while educational interventions specifically addressing nutrition care and counseling can improve self-efficacy and counseling-related outcomes [10,11,15,16]. These findings suggest that factual knowledge alone may be insufficient to develop perceived preparedness for counseling and support educational approaches involving clinical cases, practice, feedback, and simulated or real patient encounters [17].

The dietary and health-related behaviors score was independently associated with greater self-perceived counseling ability. Previous studies have similarly linked medical students’ own health behaviors and attitudes with counseling-related confidence or self-efficacy [16,18]. However, the direction of this association cannot be established, and healthier personal behaviors should not be interpreted as evidence of greater objective counseling competence.

Women had lower odds of reporting current self-perceived counseling ability than men, despite no significant difference in objective nutrition knowledge. The lower odds of reporting current counseling ability among women should not be interpreted as evidence of lower nutrition knowledge or objective counseling competence. The finding refers exclusively to self-perception and may reflect differences in confidence, self-assessment, or other unmeasured educational and social factors. This finding reflects self-perception rather than objectively assessed counseling performance and should not be interpreted as evidence of lower nutrition competence among female students.

Previous research in medical education has reported gender differences in confidence and self-assessment despite comparable objective performance [19]. Accordingly, the present result should not be interpreted as indicating lower nutrition competence among female students, and future studies should combine validated self-efficacy measures with objective assessment of counseling performance.

Overall, the findings support nutrition education that extends beyond factual knowledge. Current competency frameworks emphasize nutrition assessment, evidence-based recommendations, communication, collaborative care, and appropriate referral [5]. Practical teaching involving food-based problems, critical appraisal of information sources, and structured counseling experiences may therefore complement conventional teaching. This approach is also consistent with models suggesting that effective nutrition care depends not only on capability, but also on motivation and opportunities within clinical practice [20].

### Strengths and Limitations

This study has several strengths. It included 413 students from all six years of a medical degree, allowing nutrition-related outcomes to be examined across different stages of undergraduate training. No formal a priori sample size calculation was performed because the study was designed as an exploratory cross-sectional survey and recruitment was conducted across all six academic years during the predefined study period. Sample adequacy was additionally considered in relation to the complexity and stability of the multivariable models, including the number of observations, outcome events, model convergence, and regression diagnostics. The multidimensional assessment included general nutrition knowledge, food composition knowledge, dietary and health-related behaviors, sources of nutrition information, and self-perceived ability to provide nutrition counseling. In addition to aggregate scores, item-level analyses identified specific areas of lower performance with potential educational relevance. The analytical strategy also examined objective nutrition knowledge and self-perceived counseling ability as separate outcomes, allowing their associated factors to be explored independently. Multivariable analyses incorporated conceptually relevant covariates, robust standard errors, assessment of multicollinearity, model-performance measures, and a sensitivity analysis for missing behavioral data.

Several limitations should be considered. First, this was a cross-sectional, single-center study using convenience sampling; therefore, causal or longitudinal inferences cannot be made, and generalizability may be limited. Differences across academic stages reflect different cohorts rather than within-student progression, and the 25.7% participation rate may have introduced selection and non-response bias, particularly because students with greater interest in nutrition may have been more likely to participate.

Second, dietary and health-related behaviors, information sources, and counseling ability were self-reported and may therefore be affected by recall, social-desirability, and self-assessment biases. Counseling ability was assessed using a single item rather than a validated self-efficacy scale or objective skills assessment and should therefore be interpreted as perceived preparedness rather than demonstrated competence. Media and social networks were assessed as a single combined category, preventing separate evaluation of specific platforms or types of content and precluding assessment of frequency of exposure, credibility, or scientific quality. Therefore, the observed association cannot be attributed specifically to social media use or interpreted as evidence that these sources improve nutrition knowledge or provide accurate or educationally beneficial information. Similarly, reporting the medical curriculum, including teaching hours, specific content, teaching methods, or clinical integration, as an information source, does not quantify actual exposure to nutrition education.

Third, some explanatory variables were broad or heterogeneous, including specific dietary patterns and the composite dietary and health-related behaviors score. In addition, the multivariable models explained only part of the variability in the outcomes, and potentially relevant factors such as detailed curricular exposure, previous nutrition training, clinical experience, personal interest, communication training, self-efficacy, and opportunities to practice counseling were not directly measured. Future multicenter and longitudinal studies incorporating curricular mapping and objective assessment of counseling skills are therefore warranted.

## 5. Conclusions

Nutrition knowledge was higher among students in the later stages of medical training, and the medical curriculum and media/social networks were independently associated with higher scores. However, greater knowledge did not independently translate into greater self-perceived ability to provide nutrition counseling. These findings suggest that knowledge acquisition and counseling preparedness are distinct educational outcomes and support integrating practical, longitudinal, and competency-based nutrition training into undergraduate medical education.

## Figures and Tables

**Table 1:** Characteristics of the study population and reported sources of nutrition information.

Characteristic	n (%)
Gender	
Women	317 (76.8)
Men	93 (22.5)
Non-binary/other	3 (0.7)
Academic stage	
Years 1–2	170 (41.2)
Years 3–4	131 (31.7)
Years 5–6	112 (27.1)
Calculated body mass index category	
Underweight	23 (5.6)
Normal weight	322 (78.0)
Overweight	56 (13.6)
Obesity	12 (2.9)
Monthly household income	
<EUR 1350	25 (6.1)
EUR 1350–2100	58 (14.0)
EUR 2100–3600	143 (34.6)
>EUR 3600	104 (25.2)
Unknown/prefer not to answer	83 (20.1)
Self-perceived ability to provide nutrition counseling	
Currently capable	154 (37.3)
Not yet capable, but expects to be after graduation	200 (48.4)
Does not expect to be capable after graduation	59 (14.3)
Sources of nutrition information *	
Media and social networks	265 (64.2)
Medical degree curriculum	206 (49.9)
Family/friends/immediate social environment	176 (42.6)
Scientific literature	149 (36.1)
Individual professional advice	50 (12.1)
Non-formal education	34 (8.2)
No previous nutrition training	32 (7.7)
Other degree/training curriculum	23 (5.6)

* Participants could report up to three sources of nutrition information; percentages therefore do not sum to 100%.

**Table 2:** Bivariate associations with overall nutrition knowledge.

Variable	Category	n	Overall Nutrition Knowledge, Mean ± SD	*p*-Value
Gender ^†^	Women	317	74.7 ± 13.9	0.794
	Men	93	75.1 ± 16.1	
Academic stage	Years 1–2	170	70.8 ± 13.6	<0.001
	Years 3–4	131	75.0 ± 14.7	
	Years 5–6	112	81.0 ± 13.3	
Household income ^‡^	Low/medium–low	83	74.8 ± 15.0	0.119
	Medium–high	143	74.5 ± 13.3	
	High	104	77.5 ± 14.6	
	Unknown/prefer not to answer	83	72.5 ± 15.3	
Specific diet/dietary pattern	No	380	74.2 ± 14.4	<0.001
	Yes	33	82.9 ± 12.4	
Supplement use	No	252	73.1 ± 14.8	0.002
	Yes	161	77.6 ± 13.5	
Self-perceived counseling ability	Currently capable	154	79.1 ± 14.8	<0.001
	Not yet capable, but expects to be after graduation	200	71.7 ± 13.6	
	Does not expect to be capable after graduation	59	74.8 ± 13.8	
Medical curriculum	No	207	71.9 ± 14.6	<0.001
	Yes	206	77.9 ± 13.7	
Scientific literature	No	264	73.3 ± 14.7	0.003
	Yes	149	77.7 ± 13.6	
Media/social networks	No	148	71.8 ± 14.8	0.001
	Yes	265	76.6 ± 14.0	
Immediate social environment	No	237	77.0 ± 14.8	<0.001
	Yes	176	72.0 ± 13.4	
Professional advice	No	363	74.8 ± 14.6	0.639
	Yes	50	75.8 ± 13.4	
Non-formal education	No	379	74.9 ± 14.3	0.846
	Yes	34	75.4 ± 16.1	
No previous nutrition training	No	381	75.7 ± 14.2	<0.001
	Yes	32	65.2 ± 14.1	
Other degree/training curriculum	No	390	74.7 ± 14.5	0.230
	Yes	23	78.4 ± 12.6	

^†^ The three participants identifying as non-binary or another gender category were retained in descriptive analyses but excluded from the two-group gender comparison. ^‡^ For analytical purposes, the low (<EUR 1350) and medium–low (EUR 1350–2100) categories were combined.

**Table 3:** Multivariable linear regression for overall nutrition knowledge.

Variable	Adjusted β	95% CI	*p*-Value
Women vs. men	0.50	−2.84 to 3.85	0.769
Age, per year	0.21	−0.27 to 0.68	0.398
Years 3–4 vs. years 1–2	2.34	−1.01 to 5.69	0.171
Years 5–6 vs. years 1–2	6.07	2.43 to 9.71	0.001
Medium–high vs. low/medium–low income	0.49	−3.24 to 4.22	0.796
High vs. low/medium–low income	3.86	−0.33 to 8.05	0.071
Unknown vs. low/medium–low income	−0.49	−5.13 to 4.15	0.835
Specific diet/dietary pattern	4.50	−0.48 to 9.47	0.076
Supplement use	2.86	−0.10 to 5.83	0.058
Medical curriculum	3.42	0.29 to 6.54	0.032
Scientific literature	2.15	−1.21 to 5.52	0.210
Media/social networks	3.33	0.31 to 6.35	0.031
Immediate social environment	−2.89	−6.19 to 0.40	0.085
Professional advice	−0.01	−4.28 to 4.26	0.997
Non-formal education	0.80	−4.20 to 5.79	0.754
No previous nutrition training	−4.94	−10.98 to 1.10	0.109
Other degree/training curriculum	2.94	−2.57 to 8.45	0.296

Model n = 410. R^2^ = 0.186; adjusted R^2^ = 0.151. HC3 heteroscedasticity-robust standard errors were used. Reference categories were men, years 1–2, low/medium–low household income, and absence of each binary exposure.

**Table 4:** Multivariable logistic regression for current self-perceived ability to provide nutrition counseling.

Variable	aOR *	95% CI	*p*-Value
Women vs. men	0.28	0.16–0.49	<0.001
Age, per year	1.05	0.96–1.14	0.297
Years 3–4 vs. years 1–2	1.46	0.80–2.66	0.215
Years 5–6 vs. years 1–2	3.20	1.63–6.29	0.001
Medium–high vs. low/medium–low income	1.00	0.51–1.94	0.993
High vs. low/medium–low income	1.46	0.71–2.98	0.304
Unknown vs. low/medium–low income	1.18	0.55–2.53	0.668
Specific diet/dietary pattern	1.26	0.52–3.06	0.602
Supplement use	1.07	0.65–1.79	0.783
Dietary and health-related behaviors score, per 10 points	1.63	1.21–2.20	0.001
Medical curriculum	1.15	0.66–1.99	0.620
Scientific literature	1.43	0.84–2.44	0.185
Media/social networks	1.01	0.60–1.71	0.971
Immediate social environment	0.97	0.55–1.69	0.905
Professional advice	1.93	0.91–4.11	0.086
Non-formal education	1.66	0.71–3.88	0.243
No previous nutrition training	0.66	0.17–2.50	0.540
Other degree/training curriculum	2.55	0.88–7.44	0.086
Overall nutrition knowledge, per 10 points	1.08	0.89–1.30	0.437

* aOR, adjusted odds ratio; CI, confidence interval. Model n = 397; 148 participants reported current self-perceived counseling ability. McFadden pseudo-R^2^ = 0.179; AUC = 0.776. Reference categories were men, years 1–2, low/medium–low household income, and absence of each binary exposure.

**Table 5:** Five retained nutrition knowledge items with the lowest proportion of correct responses.

Domain	Item	Correct Response	Correct Responses, %
Food composition knowledge	Red meat has a high salt content	Disagree	22.3
General nutrition knowledge	Whole milk is nutritionally better than skimmed milk because it contains more calcium	Disagree	40.2
General nutrition knowledge	Except for corn and rice, all cereals contain traces of gluten	Disagree	48.4
Food composition knowledge	Frozen vegetables have a high salt content	Disagree	55.9
General nutrition knowledge	Using approximate portion-size techniques (e.g., the hand method) is useful for following a balanced diet	Agree	56.2

## Data Availability

Data presented in this study are available from the corresponding author upon reasonable request, subject to appropriate ethical and institutional considerations. The individual-level dataset is not publicly available due to privacy and confidentiality considerations concerning the study participants.

## References

[1] GBD 2017 Diet Collaborators (2019). Health effects of dietary risks in 195 countries, 1990–2017: A systematic analysis for the Global Burden of Disease Study 2017. Lancet.

[2] Crowley J., Ball L., Hiddink G.J. (2019). Nutrition in medical education: A systematic review. Lancet Planet. Health.

[3] Bassin S.R., Al-Nimr R.I., Allen K., Ogrinc G. (2020). The state of nutrition in medical education in the United States. Nutr. Rev..

[4] Cardenas D., Díaz G., Cadavid J., Lipovestky F., Canicoba M., Sánchez P., Álvarez L., Duarte Y., Gutiérrez Reyes J.G., Miranda de Noyola G. (2022). Nutrition in medical education in Latin America: Results of a cross-sectional survey. J. Parenter. Enter. Nutr..

[5] Eisenberg D.M., Cole A., Maile E.J., Salt M., Armstrong E., Broad Leib E., Findley T., Massa J., Albin J., Alston M. (2024). Proposed nutrition competencies for medical students and physician trainees: A consensus statement. JAMA Netw. Open.

[6] Cardenas D., Díaz G., Fuchs-Tarlovsky V., Gonzalez M.C., Carrasco F., Cano A.M.P., Bermúdez C., Maza C., Ferraresi E., Lipovestky F. (2022). Nutrition competencies for undergraduate medical education: Results of an international interdisciplinary consensus. J. Parenter. Enter. Nutr..

[7] Silva P., Araújo R., Lopes F., Ray S. (2023). Nutrition and food literacy: Framing the challenges to health communication. Nutrients.

[8] Ruani M.A., Reiss M.J., Kalea A.Z. (2023). Diet-nutrition information seeking, source trustworthiness, and eating behavior changes: An international web-based survey. Nutrients.

[9] Segado-Fernández S., Jiménez-Gómez B., Jiménez-Hidalgo P.J., Lozano-Estevan M.C., Herrera-Peco I. (2025). Disinformation about diet and nutrition on social networks: A review of the literature. Nutr. Hosp..

[10] Patel P., Kassam S. (2022). Evaluating nutrition education interventions for medical students: A rapid review. J. Hum. Nutr. Diet..

[11] Mogre V., Amoore B.Y., Gaa P.K. (2023). A scoping review of nutrition education interventions to improve competencies, lifestyle and dietary habits of medical students and residents. J. Nutr. Sci..

[12] Presa-Carrera A., Yáñez-Izquierdo E.L., Adán-Failde C.E., Vázquez-Lago J.M. (2026). Development and preliminary validation of a multidimensional questionnaire on nutrition knowledge, food composition knowledge, and dietary habits in medical students. Nutrients.

[13] von Elm E., Altman D.G., Egger M., Pocock S.J., Gøtzsche P.C., Vandenbroucke J.P. (2007). The Strengthening the Reporting of Observational Studies in Epidemiology (STROBE) statement: Guidelines for reporting observational studies. Lancet.

[14] Shaikh S., Dwivedi S., Khan M.A. (2011). Impact of learning nutrition on medical students: Their eating habits, knowledge and confidence in addressing dietary issues of patients. J. Indian Med. Assoc..

[15] Coppoolse H.L., Seidell J.C., Dijkstra S.C. (2020). Impact of nutrition education on nutritional knowledge and intentions towards nutritional counselling in Dutch medical students: An intervention study. BMJ Open.

[16] Amoore B.Y., Gaa P.K., Amalba A., Mogre V. (2023). Nutrition education intervention improves medical students’ dietary habits and their competency and self-efficacy in providing nutrition care: A pre, post and follow-up quasi-experimental study. Front. Nutr..

[17] Hauer K.E., Carney P.A., Chang A., Satterfield J. (2012). Behavior change counseling curricula for medical trainees: A systematic review. Acad. Med..

[18] Schlair S., Hanley K., Gillespie C., Disney L., Kalet A., Darby P.C., Frank E., Spencer E., Harris J., Jay M. (2012). How medical students’ behaviors and attitudes affect the impact of a brief curriculum on nutrition counseling. J. Nutr. Educ. Behav..

[19] Blanch D.C., Hall J.A., Roter D.L., Frankel R.M. (2008). Medical student gender and issues of confidence. Patient Educ. Couns..

[20] Crowley J., Ball L., Hiddink G.J. (2020). Nutrition care by primary-care physicians: Advancing our understanding using the COM-B framework. Public Health Nutr..

